# Multi-Sensor Spectral Imaging of Geological Samples: A Data Fusion Approach Using Spatio-Spectral Feature Extraction

**DOI:** 10.3390/s19122787

**Published:** 2019-06-21

**Authors:** Sandra Lorenz, Peter Seidel, Pedram Ghamisi, Robert Zimmermann, Laura Tusa, Mahdi Khodadadzadeh, I. Cecilia Contreras, Richard Gloaguen

**Affiliations:** Division “Exploration Technology”, Helmholtz Institute Freiberg for Resource Technology, Helmholtz-Zentrum Dresden-Rossendorf, Chemnitzer Straße 40, 09599 Freiberg, Germany; p.seidel@hzdr.de (P.S.); p.ghamisi@hzdr.de (P.G.); r.zimmermann@hzdr.de (R.Z.); l.tusa@hzdr.de (L.T.); m.khodadadzadeh@hzdr.de (M.K.); i.contreras@hzdr.de (I.C.C.); r.gloaguen@hzdr.de (R.G.)

**Keywords:** hyperspectral, spectral imaging, multi-sensor data, data fusion, feature extraction, support vector machine (SVM), orthogonal total variation component analysis (OTVCA), mineral exploration

## Abstract

Rapid, efficient and reproducible drillcore logging is fundamental in mineral exploration. Drillcore mapping has evolved rapidly in the recent decade, especially with the advances in hyperspectral spectral imaging. A wide range of imaging sensors is now available, providing rapidly increasing spectral as well as spatial resolution and coverage. However, the fusion of data acquired with multiple sensors is challenging and usually not conducted operationally. We propose an innovative solution based on the recent developments made in machine learning to integrate such multi-sensor datasets. Image feature extraction using orthogonal total variation component analysis enables a strong reduction in dimensionality and memory size of each input dataset, while maintaining the majority of its spatial and spectral information. This is in particular advantageous for sensors with very high spatial and/or spectral resolution, which are otherwise difficult to jointly process due to their large data memory requirements during classification. The extracted features are not only bound to absorption features but recognize specific and relevant spatial or spectral patterns. We exemplify the workflow with data acquired with five commercially available hyperspectral sensors and a pair of RGB cameras. The robust and efficient spectral-spatial procedure is evaluated on a representative set of geological samples. We validate the process with independent and detailed mineralogical and spectral data. The suggested workflow provides a versatile solution for the integration of multi-source hyperspectral data in a diversity of geological applications. In this study, we show a straight-forward integration of visible/near-infrared (VNIR), short-wave infrared (SWIR) and long-wave infrared (LWIR) data for sensors with highly different spatial and spectral resolution that greatly improves drillcore mapping.

## 1. Introduction

The usage of spectroscopic information for the evaluation, classification, and sorting of large amounts of material is an established approach in industry and mining. As speed, cost-efficiency, and reliability are crucial, the measurement setups are usually simple and highly adapted to a specific sorting problem. The algorithms often rely on binary decisions (e.g., ore–waste) based on experience-based threshold values and allow the separation into predefined object classes according to shape, size or a specific spectral property [1]. In most cases, a high spectral resolution over a large spectral range is not needed such that the acquired dataset can be limited to a few spectral bands. Hyperspectral solutions exist, but are usually focused on the ultraviolet (UV), visible/near-infrared (VNIR) or lower short-wave infrared (SWIR) range of the electromagnetic spectrum. Commercial sensor systems are available by several companies, such as LLA Instruments GmbH & Co.KG, Perception Park GmbH, Spectral Imaging Ltd. (Specim), and BK Instruments Inc. 

While these approaches are sufficient for a wide range of applications, the analysis of spectrally and spatially complex samples such as drillcores and other geological samples requires higher spectral resolution and a wider spectral range. To enable full spectrum analysis at a sufficient speed, a common approach is the integration of spectral information over a larger spatial area. This is especially advantageous if the spectral composition of the analyzed samples is locally homogenous and only large-scale compositional changes should be recorded. The most prominent spectral point sampling sensor is the HyLogger [2,3], which integrates a point spectrum over several measurements and can cover VNIR, SWIR, and long-wave infrared (LWIR). It is widely used for drill-core logging in mineral exploration [4,5,6,7]. Spectral point measurements are often combined with other parameters such as X-ray fluorescence [8], magnetic susceptibility or gamma ray attenuation [9]. However, these methods are usually slow and pose a safety issue due to the use of high energy radiation. Also, approaches to integrate spectral point sampling with RGB imaging for a 2D extrapolation have been proposed [10]. 

The spectral logging approach is often not sufficient when a more detailed analysis of mineralogical composition as well as the mapping of textures, structures, veins or local mineral associations in complex deposits is required. To add this local spatial component, higher resolution spectral mapping with a balance between coverage, speed, and cost is needed. Push broom sensors are a common solution, where hyperspectral images (HSI) can be created by moving the samples relative to the sensor(s) at constant speed. This allows for the acquisition of data from different subsequent sensors at the same time, resulting in a high throughput of material analyzed. Most commonly, single or integrated sensors covering VNIR [11] and/or SWIR [12,13,14,15,16] are used, often in combination with RGB image data. Recent studies aim at automatic vein extraction directly from the HSI to allow an interpretation of veins and structural features based on their spectral and spatial characteristics [17]. To handle the large amount of data, machine learning approaches have recently been applied to drill core HSI. Scanning electron microscopy (SEM)-based mineral analyses using the mineral liberation analysis (MLA) software of a small representative sample can be fused with HSI and extrapolated to a larger scale by defining mineralogically meaningful classes. These defined classes can then be used as training data for the automatic interpretation of a much larger sample batch [18].

The mineral mapping capabilities of VNIR and SWIR sensors are mainly limited to iron oxides and hydroxides, phyllosilicates, hydrated sulfates and rare earth element (REE)-bearing phases [19]. Mapping of important rock-forming minerals, such as quartz and feldspars, requires an extension of the spectral range to the LWIR. The currently most used hyperspectral LWIR sensor in lab-scale imaging is the Specim AisaOWL. The push broom AisaOWL uses a cooled mercury cadmium telluride (MCT) detector and is integrated as part of the Specim SisuROCK drill-core scanner setup [20]. The field of view (FOV) of the OWL is identical to other available SisuROCK sensors, which allows for a straight-forward co-registration of RGB, VNIR, SWIR, and LWIR data [21,22], however, only at a rather coarse spatial resolution (~1.6 mm in the SisuROCK setup). The Telops Hyper-Cam provides a promising alternative: Designed as a Fourier-transform infrared spectrometry (FTIR) frame imager, it allows for a distinctly finer spatial and spectral resolution at a comparable spectral sensitivity. To our knowledge, no publications on its usage for lab-scale mineralogical sample analysis or drill-core scanning exist. 

The data integration of LWIR with VNIR/SWIR data for a combined interpretation poses a challenge, mainly due to the different nature of occurring spectroscopic features. Spectral absorptions in the VNIR and SWIR are usually caused by electron transfer processes and overtones of vibrational bonds [19]. The resulting features appear as rather discrete, narrow, and wavelength-specific minima. In contrast, absorption related features in the LWIR appear usually as wide, smooth and highly overlapping reflectance maxima [23]. Commonly, VNIR/SWIR and LWIR data are interpreted separately and only a few studies describe an integrated analysis. Two types of approaches have been published; (1) the independent analysis of each dataset and subsequent integration of abundances by geologically directed logical operators or clustering [24,25]; and (2) the concurrent analysis of both datasets after wavelength-range specific absorption feature analysis [26,27] or continuous wavelet analysis [28]. 

Those apparent gaps in lab-scale multi-sensor HSI integration motivated us to investigate; (1) the integrability and value of multi-sensor datasets acquired in different experimental setups to achieve optimal conditions for the desired application; (2) the fusion of multi-sensor data for a more reliable mineral detection using sophisticated machine learning algorithms, and; (3) the evaluation of the used commercial sensors for application fields in mineral exploration. We acquired image data of a set of geological samples with diverse mineralogy, using stereo RGB imagery as well as five hyperspectral sensors with differing specifications in terms of sensor design, acquisition speed, spatial resolution, and spectral range. For validation, we acquired mineralogical information in form of SEM-MLA maps and spectral point measurements covering the complete electromagnetic spectrum in the wavelength range between 0.35 and 15.39 µm. We co-registered the datasets, preferably using automatically extracted key points. We calculated a surface model from stereo RGB data and extracted both elevation and contour information to separate sample pixels and background in all datasets. We used the orthogonal total variation component analysis (OTVCA) feature extraction technique to select the five to seven most variant features on each dataset, reducing overall data dimensionality and size. This step is not only required to reduce the overall processing time and data redundancy, but also to tackle the “Hughes Phenomenon” [29], also known as the “curse of dimensionality”. It refers to the drop of classification accuracy that is potentially caused when the number of spectral bands is increased while the number of training samples remains limited. We used the extracted features as input for support vector machine classification with radial basis function kernel (SVM-RBF) to map mineralogical classes. We exemplify this workflow with two sample subsets; (1) with spectrally pure and well-defined, but spatially highly unbalanced classes, and; (2) with spatially balanced, but spectrally mixed, variable and sparse classes. We compare the classification accuracies of several single- and multi-sensor inputs and discuss the influence of the sensor specifications on the classification outcome. 

## 2. Materials and Methods

### 2.1. Experimental Setup and Sensor Parameters 

**Figure 1:**
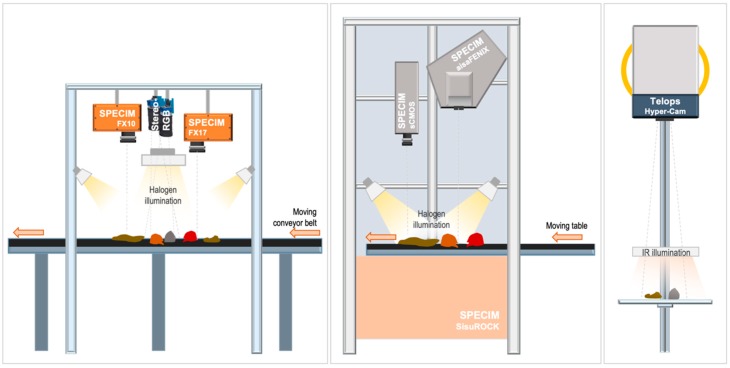
Schematic illustration of the experimental setup (not to scale). Detailed setup parameters can be found in Table 2.

Several hyperspectral sensors were used in the laboratory to verify the capability of the VNIR, SWIR, and LWIR spectral ranges in characterizing the analyzed geological samples. Additional important parameters for the applicability in the scanning of geological samples, such as spatial and spectral resolution, acquisition speed and data handling, are evaluated by comparing different sensors operating at similar wavelength ranges. The important specification and experiment parameters of the used sensors are shown in Table 1 and Table 2. In particular note, the differences in spatial resolution and field of view (FOV), which do not allow a co-registration by simple overlaying. The sensors are used in specific setups that ensure optimal conditions for each acquisition (see Figure 1 for all system setups) and demonstrate the integration of data regardless of changing external conditions. 

Such, the Specim AisaFENIX (from here on, FENIX) and the Specim sCMOS (from here on, sCMOS) are operated in a Specim SisuRock frame. Due to the smaller FOV of the sCMOS compared to the FENIX in the used setup, two separate scans are needed to cover the sample set with the sCMOS. The data of FENIX and sCMOS are thus acquired separately with different positioning of the sample box. 

The Specim FX10, FX17, and two Teledyne Dalsa RGB cameras (from here on FX10, FX17, and RGB) are used in a custom setup above a conveyor belt but could also be mounted in the SisuRock frame. In both setups, even illumination of the respective imaged area is achieved by two (SisuRock) or four (conveyor belt setup) broad-band quartz-tungsten halogen units (without protective glass cover) covering the VNIR and SWIR. For all line scanners, the images are created over time by constant linear movement of the sample table. 

The Telops Hyper-Cam (from here on, HC) is operated in a separate test stand allowing an easy adjustment of the sensor-target distance (see Figure 1). 

The scene in the HC setup is illuminated by two ceramic infrared quartz radiators, so that they provide the highest possible irradiance without a signal saturation of the sensor over the diffuse reflectors. Due to a short, but noticeable pre-heating phase, the units are left switched on during the whole experiment; the samples are only moved into the scene right before image acquisition. As the sample emissivity did not increase over the time of the measurement, we could neglect the heating of the sample. For the subtraction of ambient radiation and temperature-related sample emissivity, a “dark” scene without infrared illumination is acquired in addition to each illuminated image. A sand-blasted aluminum panel of high reflectivity (~83%) is used as a diffuse reflector to obtain the pure irradiance signal. The small FOV of the used lens and the sample-detector-distance of 1.77 m results in a high spatial resolution, but distinctly limits the image footprint. For this reason, four single images are needed to cover the sample set. 

All sensors are used at no or low binning to take advantage of the maximum deliverable spatial and spectral resolution. Exposure time and frame rate are balanced to achieve sufficient signal response at a reasonable conveyor speed. The entire measurement series is conducted under directed artificial illumination. Undirected ambient light was reduced to avoid noise by unstable or flickering radiant flux from ceiling lamps or daylight (especially at VNIR and SWIR ranges). The sensor-target-distances are primarily defined by the minimal focus distance, but also adjusted to deliver a good compromise between spatial resolution and coverage.

### 2.2. Samples Analyzed

**Figure 2:**
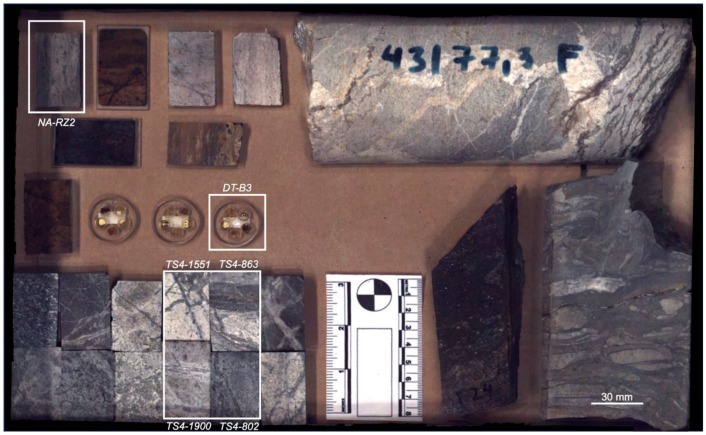
RGB photo of the analyzed sample setup, including thick- and thin-sections, drill-cores, hand specimen, and epoxy-resin embedded REE minerals. Samples analyzed in detail in the current study are marked and labeled in white.

A batch of samples with different spectral, textural, and spatial features is investigated. This comprises several cut rocks and thick-sections (~2 mm thickness) from different mineral deposits, two drill-core halves (cut face, one up and one down), one hand specimen, as well as three epoxy resin disks with rare earth element (REE)-bearing minerals from important deposits around the world (Figure 2). 

The samples are arranged and fixed on a tray to avoid any positioning changes between the single measurements. For clarity, only a subset of all samples will be depicted in the current study (marked with white frames in Figure 2). A detailed overview of those sample’s respective type, origin, and mineralogy is given in Table 3. 

### 2.3. Data Pre-processing

#### 2.3.1. Teledyne Dalsa RGB 

**Figure 3:**
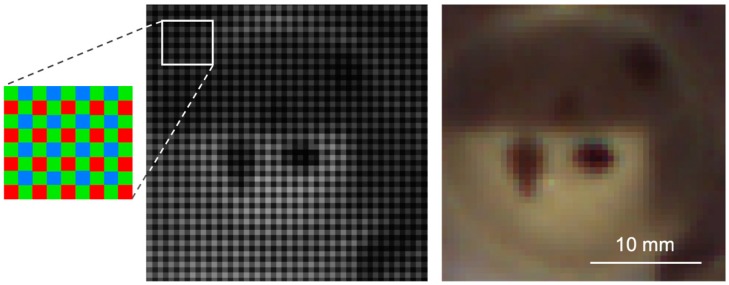
**Left**: schematic layout of a Bayer RGB matrix; **middle**: exemplary bayered RGB image, and; **right**: corresponding RGB image after debayering conversion.

True color RGB images are acquired to provide a high-resolution spatial basis for all datasets and as input for a photogrammetric surface model. Such, important information on shape, texture and topography of the observed samples can be gained. The raw images are saved as Bayer 8-bit frames, where the individual information for the red, green, and blue channel is saved side by side within a single channel in a grid-like pattern (Figure 3, left/middle). With knowledge of the color sequence, an RGB image can be calculated by respective interpolation of all red, green, and blue pixels as new separate image channels (Figure 3, right). 

**Figure 4:**
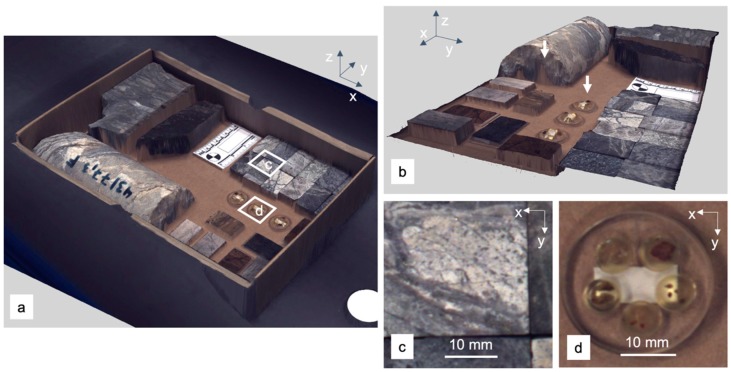
Results of the structure from motion–multiview stereo (SfM-MVS) 2.5D reconstruction. (**a**) Texturized model of the sample set, (**b**) side view to showcase artefacts at concealed regions and transparent objects (markers), (**c**,**d**) zoom to details marked in (a).

The retrieved RGB image needs to undergo subsequent white-balancing, which is achieved with a white or grey polytetrafluoroethylene (PTFE) reference target. The high frame rate and the off-nadir mounting of the two cameras returns images from the observed samples from different viewing angles. This dataset can be used to retrieve a surface model of the covered samples (Figure 4). 

We use the structure from motion–multiview stereo (SfM-MVS) [30,31] workflow implemented in Agisoft PhotoScan Professional 1.2.5. All processing parameters are adjusted to achieve the highest matching accuracy and model quality. The resulting model is cropped to the extent of the sample set. True-color orthophoto and digital surface model (DSM) are exported with a pixel size of 0.05 mm. Scene-intern markers with known spatial relations are used to reference the exported data to a true scale. For position localization, we use a non-geographical Cartesian reference system. 

#### 2.3.2. Specim HSI Sensors

The preprocessing of all push broom data is mutually similar. Before each measurement, several lines are acquired with closed shutter for dark current subtraction and with an open shutter over three PTFE calibration targets (white: Spectralon; grey and black: SphereOptics ZenithLite). The targets have known reflectance spectra averaging at >99%, 50%, and 6% reflectance in the VNIR and >95%, 46% and 6% in the SWIR, respectively. All calibration datasets are averaged along scan direction and used to convert raw digital number (DN) over radiance to reflectance. Radiance is retrieved by wavelength- and row-specific subtraction of the dark current from the raw image and subsequent multiplication with a sensor-specific calibration matrix. After this step, bad and hot pixel appear as single non-defined or infinite values on the sensor. They are automatically detected and subsequently replaced by the nearest neighbor interpolation. White, grey and black calibration target data are converted to radiance in the same manner and used in an empirical line approach to convert the image to reflectance. In the resulting reflectance image, remaining lateral illumination differences can be detected in the reflectance distribution across the calibration targets. We use these differences for a row-specific correction to achieve uniform illumination.

For the FENIX, additional geometric corrections are required to remove the effect of signal diffraction at the sensor slit and a barrel distortion across the scanning direction. The respective correction factors are provided by the manufacturer. 

#### 2.3.3. Telops Hyper-Cam

If the illumination on the sample is strong, but sample emissivity low to negligible, the reflectance, R, in LWIR data can be directly assumed from the dataset by:

(1) $$R=\frac{L_{ill}-L_{dark}}{DR_{ill}-DR_{dark}}$$

where $L_{ill}$ and $L_{dark}$ are radiance measurements with and without illumination, respectively, and $DR_{ill}$ and $DR_{dark}$ are the spectra of a diffuse reflector under the same conditions. This requires the acquisition of at least two datasets per analysis (dark and illuminated), where a diffuse reflector needs to be within the scene for each dataset. The image sets for each illumination condition are stitched to two separate image mosaics that are subsequently co-registered and subtracted from each other. The spectra $DR_{ill}$ and $DR_{dark}\;$ are calculated as mean over the imaged diffuse reflector. 

#### 2.3.4. Co-registration

The consistent nadir orientation of all hyperspectral sensors allows for a similar view on the sample set despite differing acquisition principles, spatial resolution, and FOV. This enables automatic image registration and orthorectification to the georeferenced RGB orthophoto by point detection and matching algorithms. We use scale invariant feature transform (SIFT) [32] for the extraction of robust keypoints and the fast library for approximate nearest neighbors (FLANN) [33] for point matching. The calculated reference points are then used to apply a polynomial transformation of second or third order on the respective image.

We experienced that in experiments with very dark or spectrally featureless samples sets matching failures occur and additional parameter adjustments are needed, which will prolongate the overall processing time. To increase matching accuracy, reliability, and speed, the usage of additional artificial keypoints within the scene is recommended. Especially in a combined sensor setup, high-contrast markers on the conveyor ease the registration of datasets by providing stable and easily detectable reference points. 

#### 2.3.5. Sample-background Separation and Masking

**Figure 5:**
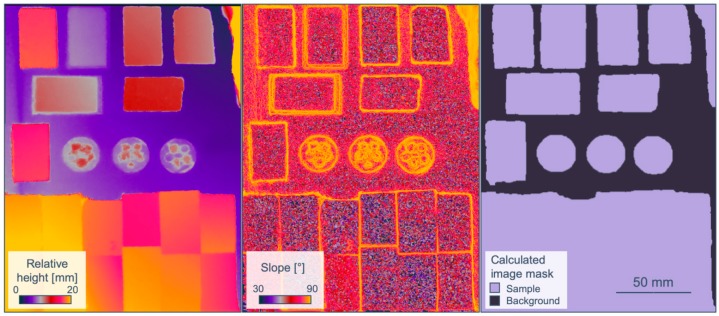
(**Left**): elevation (relative height from sample tray); (**middle**): first derivative (slope) of the elevation image; (**right**): binary mask based on both slope contours and elevation.

The separation of samples and background is a required task to both reduce data size and enhance feature extraction performance. In particular, for larger amounts of irregularly shaped samples, manual masking is not possible within a reasonable time. Automatized classification based on spectral features or reflectance intensity usually fails whenever the spectral response of the background is similar to that of the sample. This happens for example when the sample tray is dusty or with dark and featureless samples. An extraction based only on elevation information is also not reliable, as the borders of very flat samples such as thin sections can be easily mistaken for background and, vice-versa, slopes or artifacts in the background as samples (Figure 5, left). 

To account and compensate for all these, we suggest a sample-background separation based on both elevation contours and values. Both can be retrieved from the high-resolution stereo RGB data. In the current case, first a slope map is calculated from the previously provided photogrammetric DSM, indicating abrupt changes in elevation due to evident samples by increased values (Figure 5, middle). These changes can be extracted as contours, e.g., by using the approach of Suzuki and Abe [34], which is implemented in the OpenCV toolbox [35]. Subsequently, the average elevation values within each contour are compared to the expected background elevation. Whenever this value exceeds a certain threshold height, the specific contour is identified as sample. With this approach, even transparent, flat or neighboring samples are detected, while elevation artifacts or markers in the non-sample areas are ignored (Figure 5, right). The final mask is then resampled and applied to any co-registered dataset.

#### 2.3.6. Illumination Effects

The slightly off-nadir mounting of the illumination units in all measurement setups provides a uniform illumination of the investigated samples and prevents core shadows. Remaining reflectance deviations due to illumination are mostly related to cast shadows near sharp object borders, whenever one of the irradiance sources is blocked. Where two illumination sources are used, e.g., in the FENIX setup, this results in shadows along the scanning direction, whereas with four lamp units, e.g., RGB, FX10, FX17, shadows both along and across scanning direction can be observed. The manifestation of shadows within the datasets is additionally dependent on the relative position of sensor and illumination units, which results in different shadow patterns in each dataset. As the shadows are affecting mostly the sample tray and not the samples themselves, the influence of illumination differences can be neglected for this study.

#### 2.3.7. Validation

For spectral validation, all samples are analyzed at single reference points using the Spectral Evolution PSR-3500 (from here on PSR) and Agilent 4300 FTIR handheld spectrometer with diffuse reflectance cell (from here on FTIR). Specifications of each sensor are listed in Table 1. Each PSR spectrum is the result of 10 consecutive averaged measurements. A PTFE panel with over 99% reflectance in the VNIR and over 95% reflectance in the SWIR is used for reflectance conversion. Each FTIR scan is created by 32 consecutive averaged measurements and converted to reflectance using a diffuse gold standard. Using this approach, each reference point is characterized by a continuous, high-resolution reflectance spectrum ranging from 0.35 µm (Near-UV) up to 15.4 µm (LWIR). From all rock cuts and thick-sections, high-resolution mineralogical maps exist, which were derived from the SEM-MLA analysis. The MLA data are resampled to a pixel size of 0.15 mm to match the pixel size of the Teledyne RGB sensors. During the resampling, the proportion of the area for each mineral phase within one output pixel is determined and stored as pixel value in a separate channel of the output image. In this way, a datacube of separate mineral maps is created, maintaining the information of all mineral phases and allowing the visualization of the relative abundances of specific mineral phases. Subsequently, all MLA maps are geometrically referenced on the dataset to provide a 2D mineral map validation for the HSI results. The referencing is done manually. On some samples, MLA has been conducted on the counterparts of the imaged samples. Because of sample material that has been lost during the cutting and polishing process, the MLA data of these samples show slight deviations to the surface of the samples in the dataset. Especially in very heterogeneous samples this can lead to large visual differences, complicating the comparison to image-derived maps and causing false negatives during the validation. 

### 2.4. Feature Extraction and Classification

**Figure 6:**
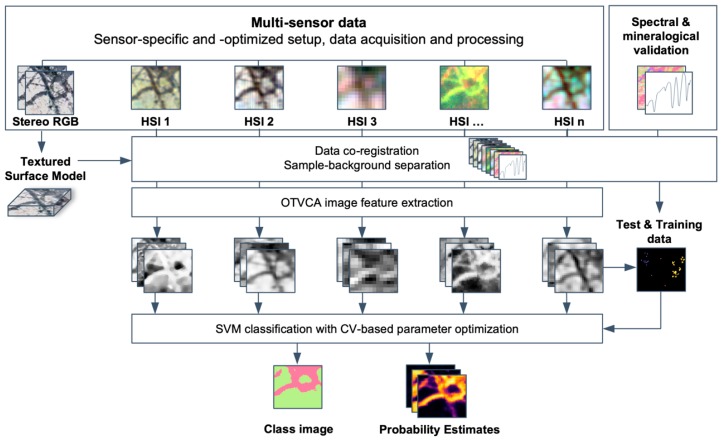
Proposed workflow for the fusion of multi-sensor HSI data using OTVCA feature extraction and SVM classification.

A schematic illustration of the proposed data integration workflow is shown in Figure 6. 

Feature extraction is conducted using the orthogonal total variation component analysis method (OTVCA [36], further extended for multi-sensor data fusion in Rasti et al. [37]). Based on the investigations conducted in Ghamisi et al. [38], OTVCA outperforms several widely-used supervised and unsupervised feature extraction approaches in terms of classification accuracies. The algorithm aims to find the best representation of a high dimensional HS input space in a low dimensional feature space by optimizing a non-convex cost-function. To preserve the spatial structure of the features, OTVCA solves a total variation (TV) penalized least square cost-function subjected to an orthogonality constraint. This constraint achieves the consideration of spatial neighborhood information during feature extraction, which is advantageous for the classification of images with fine spatial resolution having homogeneous structures. From each single-sensor dataset, around five to seven spectrally and spatially meaningful features are extracted to achieve a dimensionality near to the number of expected classes. The exact number of features is confirmed by a visual check. The resulting image features of all sensors are stacked and resampled to the input file with the finest spatial resolution (here: sCMOS). This creates a spatially highly resolved image containing the most important spectral information of each input dataset, while at the same time featuring a highly reduced data size.

Classification is conducted by using the support vector machine algorithm (SVM, using LibSVM by Chang & Lin [39]) with the radial basis function kernel on both single- and selected multi-sensor subsets of the OTVCA image feature stack. Based on the studies reported in Ghamisi et al. [40], SVM with the RBF kernel can provide accurate classification results in a stable way compared to other advanced classifiers such as backpropagation neural network, random forest, extreme learning machines, and one dimensional convolutional neural network, even if only a limited number of training samples is available. This mainly originates from its concept, which is based on margin maximization rather than statistical criteria. This makes SVM advantageous for our dataset, which is characterized by a high dimensionality of the input data due to the multi-sensor approach, a limited amount of reliable training data and mineralogically mixed (heterogeneous) classes. The optimal hyperplane parameters *C* (parameter that controls the amount of penalty during the SVM optimization) and γ (spread of the RBF kernel) have been traced in the range of γ = 2^x^ and *C* = 10^y^, with *x* in [−3, −2, −1, 0, 1, 2, 3, 4] and *y* in [−2, −1, 0, 1, 2, 3, 4], respectively, using five-fold cross-validation (compare also Ghamisi et al. [38,40]). Both the classification image as well as the probability estimates for each class are retrieved. The classification accuracies are returned as overall accuracy (OA) and average accuracy (AA). While OA returns the percentage of all true positives on the total number of reference points, AA averages the separate true positive percentages of all classes. A comparison of both values allows statements on the homogeneity of the class accuracies. A large difference between OA and AA indicates a lack in the accuracy of only one or a few classes, while similar values of OA and AA report comparable classification accuracies between all classes. 

## 3. Results

### 3.1. Spatial and Spectral Integration of the Multi-Sensor Dataset

**Figure 7:**
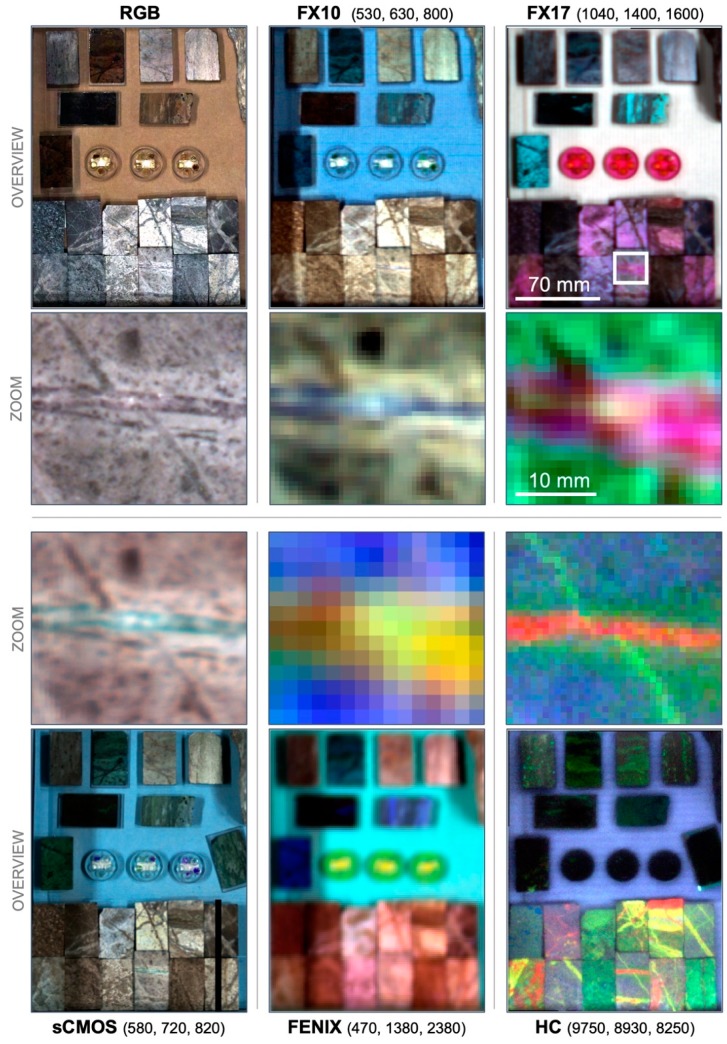
Spatial overview on the acquired datasets, shown in false color RGB (displayed wavelengths in parenthesis). For each dataset, an overview image and a zoom to the top half of sample TS4-1900 (extends marked by a white rectangle) are shown next to each other.

An overview on the processed and registered multi-sensor dataset is shown Figure 7, illustrating the large differences in spatial resolution. The co-registration of all datasets allows for the comparison of spectra retrieved with different sensors at the exact same spot. The best co-registration results are achieved if the spectral range or the spatial resolution of the base and the dataset to register are comparable, which can be used to retrieve faster and more reliable matching results. For example, due to their more comparable spatial resolution, the number of good matches between FX17 and FX10 data is much higher than between FX17 and the RGB orthophoto. In contrast, FX10 data can be matched well to the RGB base due to their overlapping wavelength ranges, and after registration serve itself as a new base for the matching of FX17 data. Thus, we are able to register all datasets fast and automatically to a common and meaningful reference space.

**Figure 8:**
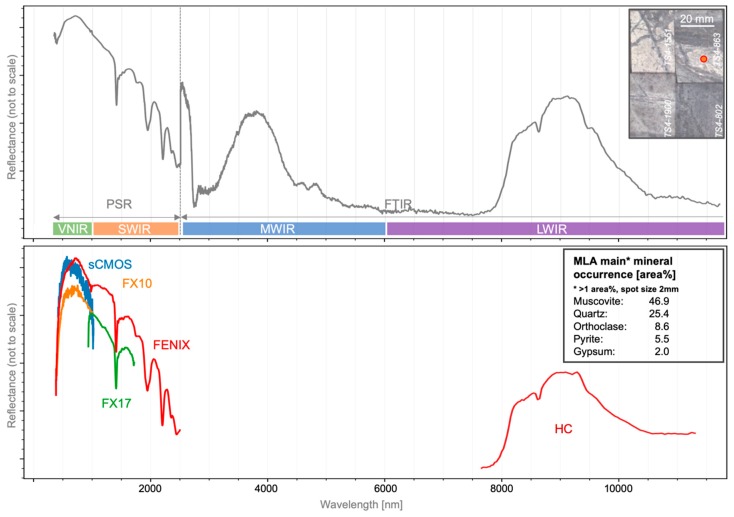
Overview on the spectra from different sensors at one validation point on sample TS4-863. **Top**: merged validation point spectra and position of the measured spot; **bottom**: image spectra and MLA information for the same spot.

A respective spectral overview is displayed in Figure 8, showcasing the covered spectral ranges of each sensor compared to the spectral point measurements and MLA information for one sample spot. All displayed spectra have been retrieved from the same spatial position. Differences in spectral shape are mainly caused by the different spatial pixel size of each sensor and slight spatial deviations of the acquisition position during the spectrometer point measurements. By extracting the MLA information for each position and spot size, the mineral interpretation of observed spectral features can be supported and validated.

**Figure 9:**
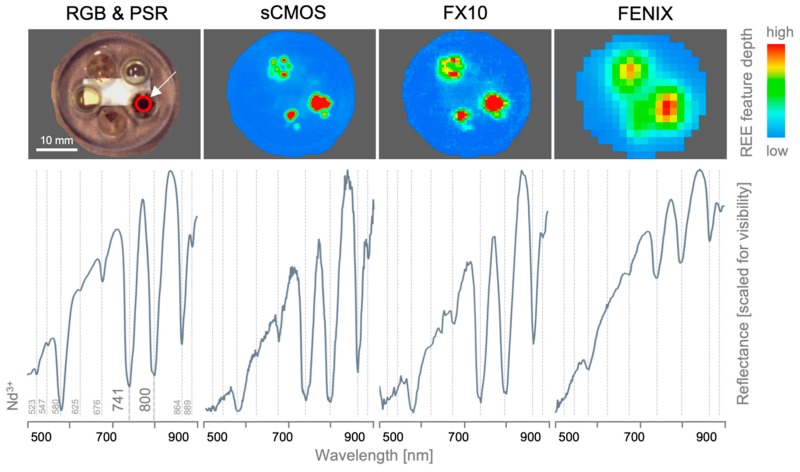
Influence of spectral and spatial resolution and sensitivity of the used VNIR sensors on the mapping of small-scale absorptions on the example of single REE grains. **Top row**: RGB image and sensor-specific REE maps (mean of the depths of the Nd^3+^-characteristic absorptions at 741 and 800 nm). **Bottom row**: single-pixel reflectance spectra of the same spot (marked with red circle) of portable spectroradiometer (PSR, outer left) and HS sensors. Positions of Nd^3+^-characteristic absorptions are marked by dashed grey lines.

To showcase the influence of the sensors’ spatial and spectral resolutions as well as sensitivities, a set of small-grained REE-bearing minerals is analyzed using all sensors covering the VNIR. The high REE content is spectrally expressed by a range of Nd^3+^-characteristic absorption features (Figure 9, lower left, absorption positions validated by Turner [41]). The depth of the most prominent absorptions at 741 nm and 800 nm can be used to create REE abundance maps for each sensor (Figure 9, top row). The influence of the spatial resolution is apparent, with highly detailed maps derived from the sCMOS, resolving even the smallest grains down to highly mixed pixels of the FENIX, where several small grains are fused into one larger object. The spectra of the largest mineral grain were extracted for each sensor at the same position and compared to a validation spectrum acquired with the PSR (Figure 9, bottom row). The sCMOS, with the highest spectral sensitivity and resolution (compare Table 1), delivers an accurate spectrum resolving even small details. Besides a decreased SNR, the spectrum is free from artefacts. The lower spectral resolution of the FX10 causes a loss in spectral detail, however, the intensity of the visible spectral features is maintained. Spectral artefacts, such as near 680 nm, complicate the reliable analysis of smaller features. Due to the large spatial pixel size and resulting increased spectral mixing, the FENIX spectrum shows a lowered depth of the visible REE features at similar overall reflectance intensity. However, the SNR and spectral resolution are sufficiently high to provide a clear spectrum depicting even smaller features. 

### 3.2. Multi-sensor Data Fusion for Image Classification 

For feature extraction and classification, two representative sample subsets with different mineralogy and spatial class distribution are selected from the sample set imaged (compare also Figure 2): sample NA-RZ2 (from here on named RZ2); and samples TS4-802, TS4-863, TS4-1551, and TS4-1900 (from here on named TS4). All single-sensor datasets are cropped to the extents of RZ2 and TS4. 

For RZ2, five mineralogically meaningful classes are defined based on MLA observations: albite, apatite, muscovite, a goethite-dominant phase (with small amounts of calcite) and a calcite-dominated matrix (with small amounts of Fe and Si). For the last two classes, the size of the individual mineral grains lies below the resolution of the MLA, thus, they need to be considered as mineralogically mixed classes. The spatial coverages of the classes are highly unbalanced, the matrix alone accounts for 75% of the sample area, while the other classes are around 5% each. As the MLA map was taken from the exact sample surface, a direct validation is possible. Thus, the MLA information is used to select the most mineralogically pure pixels (about 50% of all pixels) as test data, of which only 100 pixels for each class are excluded and used as training data (0.3% of all pixels, see Table 4). A substantially higher amount of training data cannot be achieved for this sample due to its highly unbalanced spatial distribution of classes.

For TS4, six mineralogically meaningful classes are defined: quartz, gypsum, anhydrite, muscovite, the feldspars (orthoclase, albite, labradorite), and the sulfides (pyrite, chalcopyrite). Due to the material offset between the available MLA validation maps and the imaged surface, only a visual validation is possible. Additionally, MLA is not able to distinguish gypsum and anhydrite, as the mineralogical difference is the content of lattice water. Thus, the training data of TS4 is selected manually. Beside the MLA maps, the selection is guided by characteristic features apparent in the image spectra and the analysis of validation spectra in regions uniform within the extracted OTVCA features. For each class, around 50 pixels are selected and split randomly into a test and training dataset of around 25 pixels per class each (Table 4). Compared to RZ2, all classes are approximately balanced, however, subpixel mixtures of some classes occur, in particular amongst muscovite, quartz, and feldspars. 

**Figure 10:**
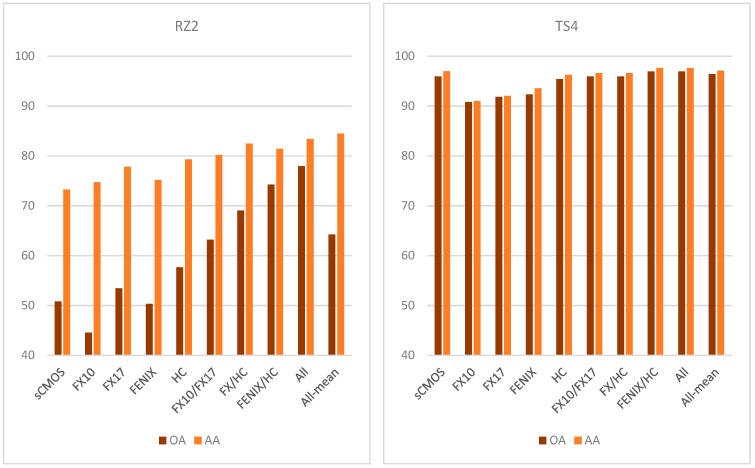
Achieved classification accuracies in sample subsets RZ2 (**left**) and TS4 (**right**) using single-sensor data, selected multi-sensor combinations as well as the complete multi-sensor dataset (labeled “All” for SVM on the complete dataset and “All-mean” for an averaging of all single-sensor SVM results). Accuracies are given as both overall accuracy (OA) and average accuracy (AA).

The achieved SVM classification accuracies for sample subsets RZ2 and TS4 are plotted in Figure 10. 

**Figure 11:**
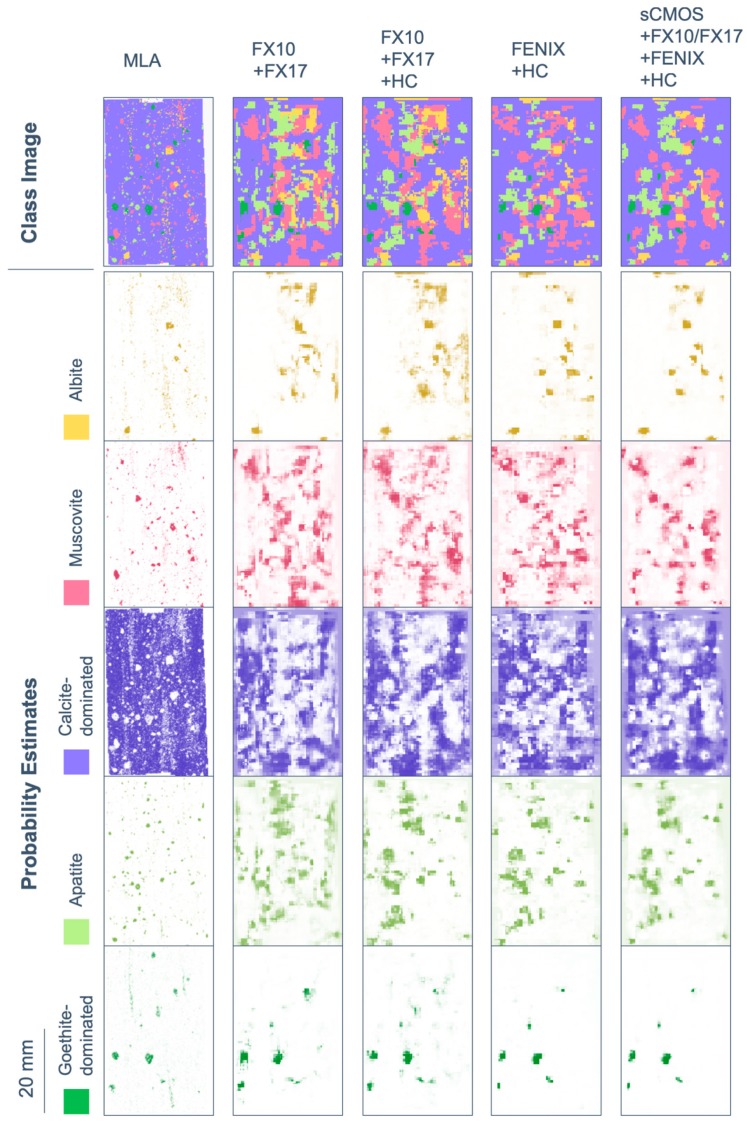
Results of SVM mapping of the five most abundant minerals and mineral groups in sample subset RZ2 using selected multi-sensor combinations, compared to the available MLA information. Shown here are both the categorized map (class image) as well as the probability estimates for each class. Probability estimates are indicated by the respective class color opacity between no opacity (0% probability) and full opacity (100% probability).

**Figure 12:**
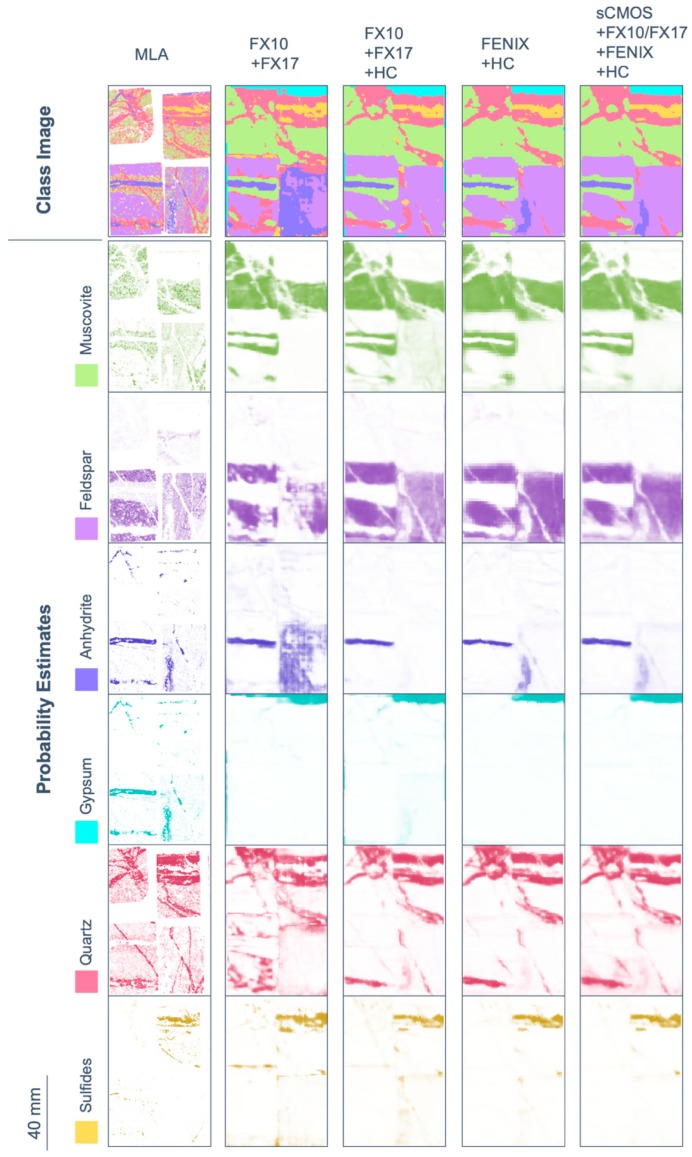
Results of SVM mapping of the six most abundant mineral classes (labeled with dominant mineral) in sample subset TS4 using selected multi-sensor combinations, visualized next to the MLA information of the sample counter pieces. Mind that gypsum and anhydrite are not separated by MLA because of their high similarity in chemical composition. Shown here are both the categorized map (class image) as well as the probability estimates for each class. Probability estimates are indicated by the respective class color opacity between no opacity (0% probability) and full opacity (100% probability).

In Figure 10, OA and AA are shown for several classification attempts, using only OTVCA features of single sensors as well as specific multi-sensor combinations. The complete multi-sensor dataset is classified in two different ways: (1) using SVM on all input bands as in the proposed workflow (labeled “All”) and (2) by majority voting of all single-sensor SVM results (labeled “All-mean”). For different sensor combinations, the respective class images and probability estimates of each class are displayed in Figure 11 for RZ2 and in Figure 12 for TS4 next to the available MLA mineral maps. 

Classification accuracy assessment of RZ2 shows a high difference between OA and AA for most single-sensor and some multi-sensor input. The main reason is the fine-grained texture and unbalanced distribution of classes in the sample. Four out of five classes occur finely distributed with grain sizes below the spatial sampling distance of most sensors used. Despite mineralogically well-defined training and test data, the related pixel spectra often contain a mixture of classes. The extents of the spatially inferior classes are, thus, often strongly exaggerated. This negatively affects the classification accuracy of the calcite-dominated class, which covers the largest part of the sample. The resulting unbalanced accuracies between the single classes become visible as increased offset between OA and AA. The fusion of the spectrally most sensitive sensors (FENIX, HC) with spatially well resolved input (sCMOS, FX sensors) retrieves the best classification accuracy, visual evaluation and class separation. It even allows for the true positive classification of object borders lying in the subpixel space of some datasets. Sample subset TS4 features a better balance between classes, however, the training data is more sparse and the classes less distinct. For example, the abundances of quartz, muscovite, and feldspar are often correlated, which generates fluent transitions between the classes labeled as quartz-, muscovite- and feldspar-dominated, respectively. However, the prior extraction of image features and corresponding assignment of training pixels allows for a proper discrimination in the classification. The classes gypsum and anhydrite, which cannot be separated in MLA analysis, are clearly distinguished in the spectral data. In general, the classification accuracies and visual validation show that the use of multi-sensor data achieves a better classification than the use of single-sensor data. Comparing FX10/FX17 and FX10/FX17/HC, the added value of LWIR data for the accurate discrimination of silicates becomes striking, especially when comparing the probability estimate maps of quartz and feldspar. However, the FX sensors can already give a fair estimation of the distribution of these classes, in particular with respect to the lack of O-Si-O stretching bonds or Al-OH, Fe-OH or Mg-OH overtones in their spectral range. Here, the advantage of prior image feature extraction accounts, as it does not limit the classification to discrete absorption features, but allows general spectral and spatial patterns to be recognized. 

Similar to sample RZ2, the fusion of spectrally meaningful data with spatially highly resolved images enabled the mapping of objects and structures lying in the subpixel scale of the coarser resolved input. Thus, the fusion of FENIX data with finer resolved HC data allows the clear separation of veins, which have a width of only half the size of a FENIX pixel. As expected, the further inclusion of spatially highly resolved VNIR and lower SWIR data (FX and sCMOS), achieved the highest classification accuracy of all tests. Visual validation confirms its classification outcome as overall best result, too. 

Both datasets show that SVM on multi-sensor data (“All”) achieves higher accuracy than averaging/majority voting of previously calculated single-sensor-SVM (“All-mean”). With multi-sensor input, a concurrent evaluation of features in several input data is possible, while unrelated information can be suppressed. In contrast, the combination of single SVM-results may give weight to data inconclusive for the evaluated class and, by that, decrease the classification accuracy. 

Cross-validation at every performed SVM has been observed as a crucial step. For example, the cross-validation accuracy for the multi-sensor dataset at TS4 can vary between 8% and 99.3%, depending on the chosen parameters. 

## 4. Discussion

The proposed workflow provides a way to fuse multi-sensor data regardless of their initial spatial and spectral characteristics. It allows for the simultaneous analysis of spectral and spatial features of one mineral class in different wavelength ranges. Thus, spatially highly resolved data can be fused with spectrally sensitive and diverse sensor information to accurately map even small-scale complex mineral structures. The precedent extraction of image features using OTVCA reduces dimensionality and data size, and by that eases the handling of multi-sensor data. Highly advantageous is the possibility to fuse information from data with very different spectroscopic properties, such as VNIR/SWIR and LWIR data. Future studies could extend the data input beyond reflectance data and include for example information from photoluminescence or Raman scattering experiments. 

The results show that data integration based on machine learning techniques allows for the selection of a smaller number of sensors required to solve a specific classification problem. The mineralogical composition of most samples justifies the definition of mixed classes, distinguishing rather spectral domains resembling characteristic mineral mixtures than single minerals. The used workflow allows to base the classification of these domains not only on distinct absorption features, but on different spectral and spatial variations. Thus, we are able to discriminate minerals that do not show specific spectral features in the currently used spectral range. For example, the FX data allowed to discriminate the quartz, feldspar, and sulfide classes that actually have no distinct spectral features in the covered VNIR and SWIR range. Even if the inclusion of the LWIR range further increased the classification accuracies for these mineral domains, the result of the FX data is promising. It shows that using advanced image processing tools, sensors with reduced spectral range can still provide a meaningful classification of main mineral domains, suggesting the use of rather low-cost and fast sensors for a first sample classification. If a more detailed analysis on selected samples is required, sensors with optimized specifications for the current mineralogy can be applied. For a successful implementation of this approach, an accurate validation is crucial. It is required to define and interpret mineralogical classes, as well as set training and test data for the classification itself. The validation can be provided by detailed mineralogical analysis of selected samples, and by the spectral characterization of possible domains using accurate point spectrometer data. 

According to the outcomes of the study, the applicability of the used HS sensors for specific tasks in lab-scale mineral mapping can be evaluated (see also Table 5).

The FENIX has the coarsest spectral resolution; however, it offers the widest spectral range and an overall sufficient spectral quality with high SNR. It is the only tested HSI sensor covering the wavelength range between 2000 and 2500 nm, which is essential for the detection of minerals containing AlOH, MgOH, FeOH or CO_3_ groups by their vibrational overtones. The FENIX is mostly not able to spatially resolve narrow veins or single mineral grains. Therefore, finely disseminated minerals of interest might not be detected when the intensities of their spectral features do not exceed the noise level of the encompassing mixed pixel spectrum. In contrast, the FENIX can provide an overview on the overall composition of the sample, distinguish lithological zones or larger veins. The data acquisition and pre-processing are quick and straight-forward and able to cover large sample batches in a reasonable time, making it an ideal tool for drill-core scanning. The combination with a sensor with higher spatial resolution has shown to partly overcome its spatial limitations and provide accurate classification of objects at subpixel size. 

The FX10 and FX17 provide a more cost-effective alternative to the FENIX camera. Of all tested sensors, they are able to acquire data at the highest speed, thus being able to characterize large amounts of material in a short time, such as ore moving on a conveyor belt. The pixel sampling in the current setup was about 1.7 (FX17) to 2.7 (FX10) times higher than those of the FENIX sensor, providing a distinctly higher amount of detail. With an adjusted measurement setup, even lower spatial sampling rates could be achieved, but at the cost of a lower area coverage. The observed SNR of the FX10 is lower than the FENIX, manifesting itself in stripy bands and noisier spectra. With a combined wavelength range of both FX sensors of 400 to 1700 nm, the amount of detectable mineral features is, additionally, fairly limited, including mostly Fe^3+^-, Fe^2+^-, OH^−^- and REE^3+^-bearing phases. Whereas in a single-sensor setup this constrains the classification accuracy, a combined interpretation of both FX datasets is able to reach reasonable classification results even for mineral classes showing no distinct absorption features within the used spectral range. Overall, the FX sensors are best suited for high-speed scanning and categorizing of samples with high spatial detail and limited spectral complexity, such as the analysis of mixed ore-waste streams. 

Similar to the FX10, the covered wavelength range of the sCMOS sensor is limited to the VNIR, however, with the fore-optics used it offered the highest spatial and spectral sampling and sensor sensitivity of all HS sensors in the current setup. Operated at full resolution, this combination of specifications results in low scanning speeds and extreme data sizes. We recommend the use in this mode for the detailed analysis of selected samples only, for which it provides an ideal tool for the accurate mapping of REE, even at low concentrations and host mineral sizes. By using image feature extraction techniques, the data size can be substantially decreased with only a small loss in spectral information; e.g., the extraction of five OTVCA features from sample set TS4 reduced the data size by the factor 45, for 15 extracted features by factor 16. The extracted components can be used as additional input for the classification of fine-grained samples. Despite the low added value for spectral interpretation, sCMOS data has shown that its spatial information crucially increases the classification accuracy of such samples. 

The HC is the only tested HSI sensor covering the LWIR range, enabling the mapping of many important rock-forming minerals such as quartz, feldspars or carbonates. Its high spectral and spatial resolution allows for a detailed mapping of small veins and mineral grains. The SNR is sufficient to retrieve clear spectra at short illumination times. Despite being designed as a frame imager, the Hyper-Cam can be also operated as quasi-line-scanner. By reducing the simultaneously acquired rows to a minimum (two to eight), frame rates of up to 30 Hz can be achieved. The respective scanning speed is reasonable for a future integration of the sensor in a joint setup with VNIR/SWIR push broom imagers. However, the high initial capital expenditure of the sensor limits its application possibilities. Due to the wide and smooth nature of mineral features in the LWIR, a narrow-band multi-spectral sensor with a similar spatial resolution might be an asset. 

## 5. Conclusions

We have shown that the integration of multi-sensor data with different spatial resolution and spectral range is feasible and highly advisable in near-field mineral exploration. Separate acquisitions allow for sensor-specific adjustments of the experimental parameters and, thus, an optimal result and a high flexibility in used sensors. Feature extraction using OTVCA enables a strong reduction in dimensionality and memory size of each input dataset while maintaining the majority of its spatial and spectral information. This is in particular advantageous for sensors with very high spatial and/or spectral resolution, but low scan speed and high data volume production. The extracted features are not bound to the occurrence of specific absorption features, but recognize any spatial or spectral patterns. Therefore, we are able to overcome differences in spectral range specific characteristics, e.g. for the fusion of VNIR/SWIR and LWIR data in combined classification approach using SVM. In parallel, the feature extraction approach enables the differentiation of classes with indistinct or mixed spectral features. These mineral domains can be discriminated, even if the most characteristic spectral features of the single minerals are not in the analyzed spectral range, reducing the number of sensors required for an overview mapping. This observation might be of additional interest for applications outside the laboratory, where the atmospheric contribution limits the observable spectral ranges and usually decreases the detectability of small spectral features.

For a more detailed spectral analysis, different multi-sensor combinations can be advantageous. A combination of sensors in the VNIR, SWIR, and LWIR range allows for a simultaneous detection of both alteration and rock-forming minerals and increases the detection reliability of certain minerals with features in different wavelength regions. The integration of very high spatial resolution data can be used to map mineralogically complex samples at a higher resolution than provided by the spectral dataset. The presented workflow allows for the mapping of mineral domains on a categorical basis and as semi-quantitative estimation of relative abundance. With sufficient validation data, a correlation between probability estimates and material abundance can be found to deliver quantified results.

Within the workflow, stereo RGB cameras revealed a cost-efficient possibility to create high-resolution spatial imagery and surface elevation models, usable for detailed sample overview, data co-registration and sample/object detection. Due to their high spatial resolution, RGB stereo data can be a potential source for surface roughness analysis, texture classification and domain extraction in the future. Similar domains might be extracted from any other spatially expressive data sources and could be included in the image classification for a clearer delineation of classes. Further promising information to integrate could originate from other surface mapping approaches beyond reflectance data, such as photoluminescence or Raman Scattering.

## Figures and Tables

**Table 1:** Overview on the sensors and their specifications used.

Sensor	Spectral Range	Spectral Res. (FWHM)	Approx. Peak-SNR	Image Size (px)	FOV	Spatial Res.*
Teledyne Dalsa C4020 (2x)	RGB (Bayer)		-	4000 × 2000 px frame	54.6°× 27.3°	0.15 mm
Specim sCMOS	VNIR: 0.40–1.00 µm	2.9 nm	170:1	2185 px line	15°	0.08 mm
Specim FX10	VNIR: 0.40–1.00 µm	5.5 nm	600:1	1024 px line	54°	0.58 mm
Specim FX17	SWIR: 0.90–1.70 µm	8 nm	1000:1	640 px line	75°	0.96 mm
Specim AisaFENIX	VNIR: 0.38–0.97 µm SWIR: 0.97–2.50 µm	3.5 nm; 12 nm	600–1000:1 1050:1	384 px line	32.3°	1.54 mm
Telops Hyper-Cam	LWIR: 1300–881 cm^−1^/7.70–11.80 µm	6 cm^−1^/ 36–76 nm	250:1	320 × 256 px frame	6.4°× 5.1°	0.62 mm
Spectral Evolution PSR-3500	VNIR: 0.35–1.00 µm SWIR: 1.00–2.50 µm	3.5 nm; 7–10 nm	600:1	Point measurement	-	~5.00 mm
Agilent 4300 FTIR	SWIR-LWIR: 4500–650 cm^−1^/2.22–15.39 µm	2 cm^−1^/ 1–47 nm		Point measurement	-	~2.00 mm

* Length of quadratic pixel.

**Table 2:** Setup parameters for each sensor used for the experiments performed in the present study.

Sensor	Sensor-Target- Distance	Exposure Time	Frame Rate	Conveyor Speed*	Binning (spat/spec) *	Data Size (150 × 240 mm)
Teledyne Dalsa C4020 (stereo)	60 cm	1 ms	8 Hz (frame)	13 cm/s	-	75 MB
Specim sCMOS	65 cm	10 ms	60 Hz	0.8 cm/s	2/1	5600 MB
Specim FX10	58 cm	4 ms	240 Hz	13 cm/s	1/2	133 MB
Specim FX17	40 cm	4 ms	140 Hz	13 cm/s	1/1	50 MB
Specim AisaFENIX	102 cm	VNIR: 14 ms SWIR: 4 ms	30 Hz	5 cm/s	VNIR: 2/2; SWIR: 1/1	37 MB
Telops Hyper-Cam	177 cm	0.25 ms	0.08 Hz (frame)	-	-	31 MB

* If applicable.

**Table 3:** Overview on the analyzed samples regarding origin, surface treatment, and mineralogy. Sample positions are marked in Figure 2.

Sample Number	Origin	Sample Treatment	Main Mineralogy ^1^/ Main REE (If Applicable) ^2^
NA-RZ2	Carbonatite, Namibia	Rock, clean cut	Cal, Ms, Ab, Ap, Fl / La, Ce, Nd
TS4-802	Copper-Gold-	Rock, clean cut	Ms, Ab, Qz, Gp, Or, Fe-Oxide, An_70_, Chm, Chl, Hbl, Ilt
TS4-863	Porphyry,	Rock, clean cut	Qz, Ms, Py, Or, Gp
TS4-1551	Romania	Rock, clean cut	Qz, Ms, Ank, Or, Gp, Hbl
TS4-1900		Rock, clean cut	Qz, Ab, Ms, An_70_, Gp, Or, Chm, Hbl, Py, Ilt
DT-B3	Miscellaneous	Embedded mineral grains, polished	Parisite/La, Ce, Pr, Nd, Sm, Y Bastnaesite/La, Ce, Pr, Nd, Sm

^1^ MLA map >1 area%, descending order, minerals abbreviated after IMA (International Mineralogical Association); ^2^ REE >0.1 wt.%, determined with electron micro probe analysis (EMPA, DT sample).

**Table 4:** Overview on important parameters of the datasets RZ2 and TS4 for classification.

Dataset	Number of Pixels	Number of Classes	Training Pixel per Class	Test Pixel per Class
RZ2	172.900	5	100	Varying (~100–85.000)
TS4	657.150	6	25	25

**Table 5:** Comparison of important performance specifications and evaluation of potential application fields for the sensors used.

Sensor Setup	Low-Cost	Speed	Spatial Detail	Detectable Minerals	Characteristic Minerals	Potential Application Fields
RGB	*****	*****	*****	*	Only shapes and textures	Photogrammetry, segmentation, texture analysis
FX	****	****	***	***	e.g., iron oxides, gypsum, micas, REEs	Fast mineral domain mapping (e.g., raw material streams)
sCMOS	***	*	*****	**	e.g., iron oxides, REEs	High spatial and good spectral resolution (e.g., low speed but accurate mineral mapping)
FENIX	**	**	*	****	e.g., clays, carbonates, REEs, micas	Mineral mapping/characterization but relatively slow (good for drillcore scanning)
HC	*	*	***	****	e.g., silicates, carbonates	Mineral mapping/characterization but relatively slow (good for drillcore scanning)
